# Serum folate predicts muscle strength: a pilot cross-sectional study of the association between serum vitamin levels and muscle strength and gait measures in patients >65 years old with diabetes mellitus in a primary care setting

**DOI:** 10.1186/s12937-016-0208-3

**Published:** 2016-10-18

**Authors:** Andrew Kien Han Wee

**Affiliations:** SingHealth Polyclinics, Marine Parade Polyclinic, Blk 80 Marine Parade Central, #01-792, Singapore, 440080 Singapore

**Keywords:** Vitamin B12, Vitamin D, Homocysteine, Fast-gait speed, Falls, Sarcopenia

## Abstract

**Background:**

Old age and diabetes mellitus are risk factors for vitamin deficiencies, weakness and falls.

Deficiencies of vitamin D, and possibly vitamin B12 and folate (which are associated with hyperhomocysteinaemia), contribute to sarcopenia. Determination of the physical effects of vitamin deficiencies in specific groups of people can help to guide public health policy with regard to vitamin supplementation.

**Methods:**

A pilot cross-sectional study was conducted to determine the association of levels of vitamin D, vitamin B12, folate and homocysteine with muscle strength, gait and fall history in 56 patients >65 years old with diabetes mellitus in primary care in Singapore.

Hand-grip and leg-quadriceps strength measures were obtained and divided by body mass index (BMI).

The timed up-and-go and Tinetti tests were used to measure gait. A history of “at least one fall in the preceding year” was obtained.

**Results:**

Vitamin B12 deficiency (<150 pmol/l) was present in 43 % of patients, folate deficiency (<13.5 mmol/l) in 20 %, hyperhomocysteinaemia (≥15.0 μmol/l) in 52 % and vitamin D deficiency (<49.9 nmol/l) in 25 %.

Levels of vitamin D, vitamin B12 and homocysteine did not significantly predict muscle strength in regression analyses.

Folate (B = 0.010, *P* < 0.01) and gender (B = 0.356, *P* < 0.001) predicted average grip strength corrected for BMI (F(2,53) = 17.74, *P* < 0.001, *R* ^2^ = 0.40)

Folate (B = 0.011, *P* < 0.05) and gender (B = 0.367, *P* < 0.001) also predicted average leg quadriceps strength corrected for BMI (F(2,53) = 9.79, *P* < 0.001, *R* ^2^ = 0.27).

Average leg strength and average leg strength corrected for BMI were both negatively associated with the risk of having fallen in the preceding year (odds ratio (OR) = 0.89, 95 % CI 0.80–0.98, *P* < 0.05 and OR = 0.12, 95 % CI 0.02–0.92, *P* < 0.05, respectively).

**Conclusions:**

The prevalence of vitamin deficiency was very high in our sample of patients >65 years old with diabetes mellitus. Folate levels were significantly correlated with grip and leg strength (with correction for BMI). Leg strength was positively correlated with gait measures and negatively correlated with a history of falls. The role of folate in muscle weakness and falls warrants further study.

## Background

Singapore has an ageing population, with an increasing prevalence of diabetes mellitus. In 2015, 11.8 % of the population was ≥65 years old [1], and a health survey conducted in 2010 found that 29.1 % of those aged 60–69 years had diagnosed diabetes mellitus [2]. In 2007, 36.3 % of patients in the primary care polyclinic in Marine Parade, where this study was conducted, were ≥65 years old [unpublished data].

Old age and diabetes are associated with the incidence and consequences of falls. A study conducted in 2011 by the Singapore Ministry of Health on 2600 community-dwelling individuals ≥60 years old in Marine Parade showed that 78.4 % suffered from at least one chronic disease, and 15 % had fallen in the 12 months preceding the survey [3]. In a prospective cohort study [4] of 63,257 Chinese men and women in Singapore, a strong dose-dependent relationship was observed between the duration of diabetes mellitus and the risk of hip fracture, leading to the suggestion that the prevention of falls should be an aspect of diabetes management. In a study involving 2847 individuals ≥65 years old presenting at the emergency department of an acute-care general hospital in Singapore, falls were the most common (13.9 %) presenting complaint [5].

Old age and diabetes mellitus are also associated with deficiencies of vitamin B12 and vitamin D.

The prevalence of vitamin D deficiency in Singapore has been reported to be 44 % in an inpatient rehabilitation unit [6], 34.5 % among patients admitted to a local hospital with hip fractures [7] and 14.3 % in an ethnic Chinese cohort 45–74 years old [8].

In a UK-based study [9], the prevalence of vitamin B12 deficiency was ~10 % in individuals 65–74 years old and ~20 % in those ≥75 years old. Vitamin B12 deficiency is common in patients with diabetes mellitus because of its association with the first-line therapy, metformin [10, 11].

In a cross-sectional study of 203 outpatients with type 2 diabetes mellitus at a US primary care clinic [12], the prevalence of metabolically confirmed vitamin B12 deficiency was 22 %, and patients receiving metformin had a significantly lower serum level of vitamin B12 than those not receiving metformin (314.4 pmol/l versus 389.3 pmol/l; *P* = 0.012).

In a cross-sectional study of 608 patients with diabetes attending the Marine Parade Polyclinic’s Family Physician (FP) Clinic, we found that one in four had a vitamin B12 deficiency of <150 pmol/l [unpublished data].

Vitamin D deficiency has a well-documented association with sarcopenia, muscle weakness and falls [13–15], and the role of vitamin D in the regulation of epigenetic DNA methylation is currently being investigated [16]. However, many of the falls that occur with old age and diabetes mellitus could also be the result of deficiencies of vitamin B12 and folate. Vitamin B12 deficiency can cause neuropathy that can lead to impaired balance and proprioception, along with amyotrophy and subsequent sarcopenia, all of which can contribute to falls [17]. Vitamin B12 deficiency can also lead to depression with consequent psychomotor retardation, contributing to subsequent falls [18, 19]. Vitamin B12 deficiency has also been associated with frailty [20], which is characterised by reductions in weight, grip strength, endurance, physical activity and walking speed, which could again contribute to falls [21]. Elevation of serum levels of homocysteine (a proxy for deficiencies in vitamin B12 and folate) could, in turn, cause neuro-cardiovascular complications, such as cognitive decline and stroke [22], that could predispose to falls. Homocysteine permanently degrades the molecular structural integrity of collagen, elastin and proteoglycans, causing predisposition to muscular–skeletal mechanical instability, osteoporosis, falls and fractures [23–25]. The results of a study conducted in Singapore [26] suggest that low levels of folate and high levels of homocysteine are associated with physical and functional decline, as measured by Performance-Oriented Mobility Assessment (POMA) and Instrumental Activities of Daily Living (IADL) scores.

Apart from a few case reports [27–32], no published studies have directly linked deficiencies of vitamin B12 and folate to the occurrence of falls.

The aim of this pilot study was to identify any association between muscle strength and gait and balance assessments and levels of serum vitamin B12 and folate in patients >65 years old with diabetes mellitus in a primary care polyclinic in Singapore.

## Methods

### Study population

Ethics approval was obtained from SingHealth Centralised Institutional Review Board and all participants provided written informed consent.

Participants were patients >65 years old and diagnosed with diabetes mellitus for ≥1 year at the Marine Parade polyclinic, which provides public primary health care to the socio-economically heterogeneous south-eastern region of the city-state of Singapore. A convenience sampling strategy was used where subjects were recruited as they presented at the clinic for treatment related to diabetes mellitus. They were referred for recruitment by doctors or nurses after diabetic retinal photography, diabetic foot screening or clinic consultations for diabetes mellitus. Disproportionate stratified sampling by gender was used to ensure equal numbers of male and female participants, and 56 patients (28 male and 28 female) were recruited. A total of 200 patients were excluded at the pre-screening phase because of recent acute illness, malignancy, severe neuromuscular disorders (such as major stroke, Parkinson’s disease, inability to walk independently despite assistive devices), inconsistent vitamin supplementation in the preceding year, illiteracy or inability or refusal to give informed consent. Regular patients of the Principal Investigator were also excluded.

### Study phases

The first phase was performed in 2012 with the recruitment of 11 patients, and provided provisional data to enable calculation of the sample size for the second phase. The second phase was performed in 2014 with the recruitment of an additional 45 patients, and involved a change in the device for measuring lower-limb strength, and the inclusion of a swaymeter to assess balance via body sway. Serum albumin and total protein were also measured in the second phase.

### Measurements

Each patient was interviewed and assessed (without fasting) in one session by the Principal Investigator and a trained Research Assistant. Overnight fasting blood samples were then obtained the following day and analysed by the Singapore General Hospital Laboratory.

### Laboratory measurements

Serum vitamin B12 and folate were analysed by Access Immunoassay Systems 33000 and A98032, respectively (Beckman Coulter, Brea, CA, USA). Homocysteine was analysed with the ARCHITECT Homocysteine Assay 1L71 (Abbott, Wiesbaden, Germany). 25-OH-vitamin D was measured with the 25-Hydroxyvitamin D ^125^I RIA Kit (68100E; DiaSorin Inc, Stillwater, MN, USA). Serum albumin and total protein were measured with the ALB2: ACN 8413 and TP2: ACN 8678 in vitro tests, respectively, on a cobas® c 701/702 analyser (Roche Diagnostics, Mannheim, Germany).

Vitamin B12 deficiency and elevated homocysteine (hyperhomocysteinaemia) were defined as <150 pmol/l [17, 18, 33, 34] and ≥15.0 μmol/l, respectively [35]. Folate deficiency was defined as <13.5 nmol/l based on the laboratory’s chosen definition, vitamin D deficiency as <49.9 nmol/l and vitamin D insufficiency as ≥49.9 nmol/l and <74.1 nmol/l [13].

### Physical performance tests to assess muscle strength, gait and balance

We used the POMA scoring system to assess performance [36, 37], which has been shown to accurately assess impairments in gait and balance that lead to increased fall risk [38]. Timed up-and-go (TUG) [39] and 6-m fast-gait-speed tests were also performed. The TUG test measured the time for barefooted patients sitting in a standard armchair to get up and walk around a mini traffic cone 3 m away and then to walk back to sit in the chair. The 6-m fast-gait-speed test assessed the time needed to walk 6 m at maximum speed [40, 41].

Leg strength was measured with the patient seated on a chair with hips and knees flexed at 90°. A foam-padded dynamometer was placed on the shin 10 cm above the lateral malleolus, and the patient exerted a maximal extension force on the pad. For the 11 patients of the first phase of this study, measurements were made with a factory-calibrated Lafayette hand-held dynamometer. However, the reliability of this device is affected by the strength of the patient [42], and its use is physically strenuous and has a risk of strain injury. A calibrated stationary dynamometer was obtained from the group of Professor Stephen R. Lord at Neuroscience Research Australia (Sydney, Australia) for leg-strength measurement in the remaining 45 patients of the second phase.

Leg quadriceps strength was measured in kilogram-force (kgf; ~9.8 N per 1 kgf). The dominant and non-dominant legs were assessed three times each with 10–20 s between each maximal extension effort. The mean strength for both legs and the mean strength and peak performance for each leg were obtained. A separate measure was obtained by dividing mean strength by body mass index (BMI), to correct for body habitus.

Hand-grip strength in both the dominant and non-dominant arms was measured with the Jamar® grip-strength dynamometer (Sammons Preston Rolyan, Bolingbrook, IL, USA) [43–45]. The patient was seated, with shoulders adducted and neutrally rotated, and elbow flexed at 90°, with the forearm in neutral position and the wrist between 0 and 30° of dorsiflexion and between 0 and 15° of ulnar deviation. Mean strength for both forearms and mean strength and peak performance of each forearm were obtained. A separate measure was obtained by dividing mean strength by BMI.

A Lord swaymeter (with foam) was obtained from the Lord group at Neuroscience Research Australia, for assessment of the 45 patients in the second phase of the study. This device provides valid and reliable measurements for assessing fall risk in a range of health-care settings [46], by determining anterior–posterior and lateral sway displacements, with a 40 cm rod attached to the patient’s waist on one end and mounted with a pen on the opposite end. The pen records the sway on graph paper. Patients had their balance tested three times with each of the four combinations of having both eyes open or shut and standing on the floor or on foam. The sway area, which is a product of the anterior–posterior displacement and the lateral displacement after 30 s of standing, was measured for each attempt, and the mean of each set of three measurements was determined.

These measurement tools were chosen also because of their portability, validation, reliability, simplicity of use and potential for future clinical applications in the primary health-care setting, where high loads of patients are the norm, and quick and reliable assessment tools are required for risk stratification.

### Other variables

Other information that was collected included physical characteristics and history of chronic disease, information on medication and lifestyle (Table 1). Data were collected from medical records on glycosylated haemoglobin (HbA1c, measured with the A1-W3: ACN 881 in vitro test on a cobas® analyser), LDL cholesterol (from the Friedewald equation, with Total Cholesterol measured with CHO2I: ACN 8798 and CHO2A: ACN 8433 in vitro tests, HDL Cholesterol measured with the HDLC3: ACN 8435 in vitro test, Triglycerides measured with the TRIGL: ACN 8781 in vitro test; cobas® analyser), alanine aminotransferase (ALT, ALTL: ACN 8685 in vitro test; cobas® analyser) and estimated creatinine clearance (from the Cockcroft–Gault equation, with creatinine measured with the CREJ2: ACN 8690 in vitro test; cobas® analyser). These measurements were taken from the most recent laboratory tests performed on all patients as part of routine diabetic care within the preceding year.

**Table 1:** Baseline characteristics of the 56 patients according to gender and folate

	Women (*n* = 28) [*n* (%) or mean ± SD]	Men (*n* = 28) [*n* (%) or mean ± SD]	*P* value	Folate deficiency (<13.5 nmol/l) (*n* = 11) [*n* (%) or mean ± SD]	Folate normal (≥13.5 nmol/l) (*n* = 45) [*n* (%) or mean ± SD]	*P* value
Vitamin B12 level (pmol/l)	205 ± 136	200 ± 103	ns	224 ± 142	197 ± 114	ns
Vitamin B12 deficiency (<150 pmol/l)	13 (46 %)	11 (39 %)	ns	5 (45 %)	19 (42 %)	ns
Folate level (nmol/l)	21.8 ± 10.8	21.1 ± 9.3	ns	10.8 ± 2.1	24.0 ± 9.5	<0.001
Folate deficiency (<13.5 nmol/l)	7 (25 %)	4 (14 %)	ns	11 (100 %)	0 (0 %)	<0.001
25-OH-vitamin D level (nmol/l)	63.6 ± 17.7	63.1 ± 20.5	ns	59.2 ± 25.5	64.5 ± 17.3	ns
25-OH-vitamin D deficiency (<49.9 nmol/l)	9 (32 %)	5 (18 %)	ns	4 (36 %)	10 (22 %)	ns
Homocysteine level (μmol/l)	16.8 ± 9.5	16.9 ± 6.4	ns	17.4 ± 6.9	16.8 ± 8.3	ns
Hyperhomocysteinaemia (≥15.0 μmol/l)	14 (50 %)	15 (54 %)	ns	7 (64 %)	22 (49 %)	ns
Ethnicity (*n* and % of respective column group)						
Chinese	24 (85 %)	19 (68 %)	ns	8 (73 %)	35 (78 %)	ns
Malay	2 (7 %)	1 (4 %)	ns	0 (0 %)	3 (7 %)	ns
Indian	1 (4 %)	4 (14 %)	ns	3 (18 %)	3 (7 %)	ns
Eurasian and Others	1 (4 %)	4 (14 %)	ns	1 (9 %)	4 (9 %)	ns
Age (years)						
Overall mean age	72 ± 5	71 ± 4	ns	72 ± 5	71 ± 5	ns
65–69 years old (*n* and % of respective column group)	10 (36 %)	13 (46 %)	ns	4 (36 %)	19 (42 %)	ns
70–74 years old (*n* and % of respective column group)	11 (39 %)	9 (32 %)	ns	5 (46 %)	15 (33 %)	ns
75–79 years old (*n* and % of respective column group)	4 (14 %)	5 (18 %)	ns	1 (9 %)	8 (18 %)	ns
80–84 years old (*n* and % of respective column group)	3 (11 %)	1 (4 %)	ns	1 (9 %)	3 (7 %)	ns
Height (m)	1.52 ± 0.05	1.65 ± 0.07	<0.001	1.55 ± 0.11	1.59 ± 0.09	ns
Weight (kg)	55.6 ± 9.0	67.9 ± 10.2	<0.001	64.4 ± 14.4	61.1 ± 10.6	ns
BMI (kg/m^2^)	24.2 ± 4.0	24.9 ± 3.78	ns	26.6 ± 3.5	24.1 ± 3.8	ns
History of chronic disease						
Hypertension	24 (85 %)	21 (75 %)	ns	10 (91 %)	35 (78 %)	ns
Hyperlipidaemia	26 (93 %)	27 (96 %)	ns	11 (100 %)	42 (93 %)	ns
Ischaemic heart disease	5 (19 %)	7 (25 %)	ns	1 (11 %)	11 (24 %)	ns
Stroke	3 (11 %)	0 (0 %)	ns	1 (9 %)	2 (4 %)	ns
Cancer (exclusion criterion)	0 (0 %)	0 (0 %)	ns	0 (0 %)	0 (0 %)	ns
Chronic obstructive pulmonary disease	0 (0 %)	0 (0 %)	ns	0 (0 %)	0 (0 %)	ns
Number of chronic medications (excluding supplements)	5.8 ± 1.6	6.0 ± 2.4	ns	6.8 ± 2.0	5.7 ± 2.0	ns
Vitamin–mineral supplementation						
Supplementation of any type	21 (75 %)	13 (46 %)	<0.05	7 (64 %)	27 (60 %)	ns
Supplementation containing vitamin B12	4 (14 %)	3 (11 %)	ns	4 (36 %)	3 (7 %)	<0.05
Supplementation containing folate	3 (11 %)	2 (7 %)	ns	2 (18 %)	3 (7 %)	ns
Supplementation containing vitamin D	12 (43 %)	4 (14 %)	<0.05	5 (45 %)	11 (24 %)	ns
Supplementation containing calcium	13 (46 %)	5 (18 %)	<0.05	5 (45 %)	13 (29 %)	ns
Taking proton-pump inhibitor	6 (21 %)	7 (25 %)	ns	4 (36 %)	9 (20 %)	ns
Taking Histamine H_2_ antagonist	0 (0 %)	3 (11 %)	ns	1 (9 %)	2 (4 %)	ns
Taking metformin	25 (89 %)	27 (96 %)	ns	11 (100 %)	41 (91 %)	ns
Metformin duration >1 year	24 (85 %)	25 (89 %)	ns	11 (100 %)	38 (84 %)	ns
Metformin 1-year cumulative dose (g/year)	543 ± 420	538 ± 311	ns	684 ± 250	505 ± 318	ns
Metformin latest daily dose (g/day)	1.49 ± 0.84	1.57 ± 0.87	ns	1.82 ± 0.73	1.46 ± 0.87	ns
Taking sulphonylureas	15 (54 %)	18 (64 %)	ns	7 (64 %)	26 (58 %)	ns
Taking dipeptidyl peptidase-4 inhibitors	3 (11 %)	4 (14 %)	ns	2 (18 %)	5 (11 %)	ns
Taking insulin	3 (11 %)	2 (7 %)	ns	0 (0 %)	5 (11 %)	ns
Smoking	1 (4 %)	4 (14 %)	ns	0 (0 %)	5 (11 %)	ns
Consumption of alcohol at least 1 unit per week	0 (0 %)	8 (29 %)	<0.01	2 (18 %)	6 (13 %)	ns
Self-reported sleep per day (h)	7.2 ± 1.6	6.8 ± 1.0	ns	6.7 ± 1.2	7.0 ± 1.4	ns
HbA1c (%)	7.3 ± 0.6	7.2 ± 0.7	ns	7.2 ± 0.9	7.3 ± 0.6	ns
^a^Estimated creatinine clearance (ml/min) (Cockcroft–Gault)	62.0 ± 19.8	68.0 ± 25.1	ns	70.0 ± 27.1	63.8 ± 21.5	ns
^a^LDL cholesterol level (mmol/l)	2.25 ± 0.61	2.18 ± 0.79	ns	2.59 ± 0.97	2.13 ± 0.60	ns
Total protein (g/l) [for patients 12–56 only; 22 male, 23 female]	72.3 ± 3.0	71.0 ± 3.8	ns	72.7 ± 3.4	71.5 ± 3.5	ns
Total albumin (g/l) [for patients 12–56 only; 22 male, 23 female]	45.0 ± 2.4	45.4 ± 2.0	ns	44.7 ± 1.3	45.2 ± 2.4	ns
Neurothesiometer VPT (V) (average of both feet)	13.21 ± 5.73	13.55 ± 6.12	ns	See Table 3	See Table 3	-
Average lower-limb strength (kgf) (average of both limbs, three attempts per limb)	17.14 ± 4.82	26.79 ± 11.78	<0.001	See Table 3	See Table 3	-
Average lower limb strength/BMI (m^2^)	0.73 ± 0.24	1.09 ± 0.41	<0.001			
Average grip strength (kgf) (average of both hands, three attempts per hand)	16.22 ± 3.29	25.54 ± 7.78	<0.001	See Table 3	See Table 3	-
Average grip strength/BMI (m^2^)	0.69 ± 0.19	1.04 ± 0.33	<0.001			
6-m fast-gait speed (m/s) (average of two trials)	1.02 ± 0.27	1.17 ± 0.28	ns	See Table 3	See Table 3	-
Timed up-and-go test (s) (average of two trials)	11.36 ± 5.72	9.53 ± 2.72	ns	See Table 3	See Table 3	-

Values are the mean ± SD or number (%) unless otherwise indicated

Pearson chi-square test and Fisher’s exact test (where appropriate) were used for categorical variables and *t*-test was used for continuous variables

For 25-OH-vitamin D, divide by 2.496 to convert from nmol/l to μg/l [68]. For vitamin B12, divide by 0.738 to convert from pmol/l to pg/ml [68]

For folate, divide by 2.266 to convert from nmol/l to μg/l [68]. For homocysteine, divide by 7.397 to convert from μmol/l to ml/l [68]

*Abbreviations*: *BMI* body mass index, *LDL* low-density lipoprotein, *ns* not significant, *SD* standard deviation, *VPT* vibration perception threshold

^a^Obtained from case records

Other tests were administered, including the Michigan Neuropathy Screening Instrument (MNSI© University of Michigan, 2000), for assessment of distal symmetrical peripheral neuropathy. The Horwell® Neurothesiometer [47], which tests the vibration perception threshold (VPT) of the tip of the big toe (from 0 to 50 V), was applied to both feet. The Semmes–Weinstein Monofilament Test [48, 49], the Barthel Index of Activities of Daily Living (BADL) [50–52] and the IADL [53] were also used.

Patients were also asked if they had fallen at least once in the preceding year. The definition of a fall was taken as “an unintentional change in position resulting in coming to rest on the ground or another lower level, and not as a result of a major intrinsic event (e.g., stroke, syncope) or overwhelming hazard (e.g., car accident)” [46].

### Sample-size calculation

There has hitherto been no similar study associating levels of vitamin B12 and folate with muscle strength, gait and balance measures in older people with diabetes mellitus in Singapore. Thus, to ascertain an effect size, provisional analysis of the first 11 patients showed a lower vitamin B12 level to be associated with a longer time to complete the TUG test with a Pearson correlation of 0.34. With a type I error of 0.05 and power of 0.8, a total of 66 patients should have been recruited to validate this level of correlation. However, a sample size of 56 patients was chosen, as a reasonable number for a pilot study. This number of patients would still yield significant results at a Pearson correlation of 0.37 or better.

In the first 11 patients, a high level of correlation was seen between the average grip strength (corrected for BMI) and serum folate (*r* = 0.85) and between the average corrected leg strength and serum folate (*r* = 0.71). Moreover, serum folate per se strongly predicted average corrected leg strength (B = 0.021, *P* < 0.05) and average corrected grip strength (B = 0.022, *P* < 0.01) in bivariate analysis for these 11 patients. However, these stronger correlations were not used for the calculation of sample size, which was instead based on the more conservative approach.

### Statistical analysis

Group comparisons for categorical and continuous variables were performed using chi-square tests, t-tests and ANOVA where appropriate. Fisher’s exact test was used to reconfirm any significant results from a chi-square test because of the small sample size. When the patient-count per cell was less than five, only Fisher’s exact test was used. Serum levels of vitamin B12, folate, homocysteine and 25-OH-vitamin D were also split into high versus low values based on accepted cut-offs, for further analysis.

Associations with muscle strength, gait and balance measures were also assessed with multivariable linear and logistic regression methods in which serum vitamin B12, folate, homocysteine and 25-OH-vitamin D were analysed as continuous predictive variables. Potential interactions between these predictive variables were explored by adding multiplicative interaction terms. Variance inflation factors were analysed after regression analyses to ensure that collinearity did not exist, because homocysteine shares the same metabolic pathway as vitamin B12 and folate.

The mean scores for the history and physical assessments of the MNSI, neurothesiometer readings and BADL were analysed for difference using t-tests. In addition, the results of the BADL (maximum score 20), IADL (maximum score 8), POMA–balance (POMA B; maximum score 16) and POMA–gait (POMA G; maximum score 12) were separated into those with and without the maximum score, and differences were analysed using chi-square and Fisher’s exact tests, as appropriate.

Physical measurements for muscle strength, gait and balance, BADL, IADL, tests for neuropathy and fall history were analysed as dependent variables. Dependent variables were analysed as continuous, categorical or ordinal variables, as appropriate. Characteristics that could affect physical performance measures (the presence or absence of smoking and/or alcohol consumption, estimated creatinine clearance, serum total protein, serum albumin, ALT, age, gender, self-reported sleep, number of chronic medications) were also explored as predictive variables in various models. Predictive variables were first individually paired with each dependent variable and analysed for correlation. Regression models were then analysed using forward and backward selection. To prevent overfitting because of the small sample size, only two parsimonious models are described (Table 2), based on statistical significance, the aims of this study and biological plausibility. Standardized and unstandardized regression coefficients (β and B, respectively), corresponding standard errors (SE) and adjusted *R* ^2^ are presented, along with F (the ratio of mean regression sum of squares to mean error sum of squares, shown with the number of degrees of freedom in the numerator and the denominator).

**Table 2:** Regression analyses of associations of variables with leg strength, grip strength, fast-gait speed and timed up-and-go (TUG) times

Determinants of strength measures (leg and grip)	Regression of average leg strength/BMI						Regression of average grip strength/BMI					
	Model 1, F(6, 49) = 3.30, *P* < 0.01			Model 2, F(2, 53) = 9.79, *P* < 0.001			Model 1, F(6, 49) = 6.25, *P* < 0.001			Model 2, F(2, 53) = 17.74, *P* < 0.0001		
	*R* ^2^ = 0.29, adjusted *R* ^2^ = 0.20			*R* ^2^ = 0.27, adjusted *R* ^2^ = 0.24			*R* ^2^ = 0.43, adjusted *R* ^2^ = 0.36			*R* ^2^ = 0.40, adjusted *R* ^2^ = 0.38		
	β	B (SE)	*P*	β	B (SE)	*P*	β	B (SE)	*P*	β	B (SE)	*P*
Gender (men = 1, women = 0)	0.4450	0.3600 (0.0982)	0.001	0.4537	0.3670 (0.0950)	<0.001	0.5406	0.3451 (0.0691)	<0.001	0.5573	0.3558 (0.0679)	<0.001
Age	−0.0727	−0.0065 (0.0117)	ns	-	-	-	−0.1792	−0.0127 (0.0082)	ns	-	-	-
Folate	0.1926	0.0078 (0.0057)	ns (0.173)	0.2681	0.0109 (0.0048)	0.026	0.3084	0.0099 (0.0040)	0.017	0.3194	0.0102 (0.0034)	0.004
Vitamin B12	−0.0868	−0.0003 (0.0005)	ns	-	-	-	−0.0138	−0.00004(0.0003)	ns	-	-	-
Vitamin D	0.1036	0.0022 (0.0028)	ns	-	-	-	−0.0076	−0.0001(0.0020)	ns	-	-	-
Homocysteine	−0.0774	−0.0039 (0.0067)	ns	-	-	-	0.0515	0.0021(0.0047)	ns	-	-	-
Determinants of speed measures (gait speed)	Regression of gait speed on average leg strength						Regression of gait speed on average leg strength/BMI					
	Model 1′, F(5, 50) = 4.67, *P* <0.05			Model 2′, F(2, 53) = 11.89, *P* < 0.001			Model 1’, F(5, 50) = 6.09, *P* < 0.001			Model 2’, F(2, 53) = 16.00, *P* < 0.0001		
	*R* ^2^ = 0.32, adjusted *R* ^2^ = 0.25			*R* ^2^ 0.31, adjusted *R* ^2^ = 0.28			*R* ^2^ = 0.38, adjusted *R* ^2^ = 0.32			*R* ^2^ = 0.38, adjusted *R* ^2^ = 0.35		
	β	B (SE)	*P*	β	B (SE)	*P*	β	B (SE)	*P*	β	B (SE)	*P*
Average leg strength	0.4546	0.0126 (0.0330)	<0.001	0.4655	0.0129 (0.032)	<0.001	-	-	-	-	-	-
Average leg strength/BMI	-	-	-	-	-	-	0.5291	0.3661 (0.0801)	<0.001	0.5347	0.3699 (0.0755)	<0.001
Age	−0.2979	−0.0185 (0.0760)	0.018	−0.2788	−0.0174 (0.0071)	0.018	−0.2541	−0.0158 (0.0073)	0.035	−0.2491	−0.0155 (0.0068)	0.026
Vitamin B12	−0.0269	−0.0006 (0.0003)	ns	-	-	-	0.0097	0.00002 (0.0003)	ns	-	-	-
Vitamin D	0.0848	0.0013 (0.0018)	ns	-	-	-	0.0448	0.0007 (0.0017)	ns	-	-	-
Homocysteine	0.0044	0.0002 (0.0043)	ns	-	-	-	0.0149	0.0005 (0.0041)	ns	-	-	-
Determinants of speed and balance measures (TUG)	Regression of TUG on average leg strength						Regression of TUG on average leg strength/BMI					
	Model 1', F(5, 50) = 3.20, *P* < 0.05			Model 2', F(2, 53) = 7.57, *P* < 0.01			Model 1', F(5, 50) = 4.24, *P* < 0.01			Model 2', F(2, 53) = 10.38, *P* < 0.001		
	*R* ^2^ = 0.24, adjusted *R* ^2^ = 0.17			*R* ^2^ 0.22, adjusted *R* ^2^ = 0.19			*R* ^2^ = 0.30, adjusted *R* ^2^ = 0.23			*R* ^2^ = 0.28, adjusted *R* ^2^ = 0.25		
	β	B (SE)	*P*	β	B (SE)	*P*	β	B (SE)	*P*	β	B (SE)	*P*
Average leg strength	−0.3487	−0.1555 (0.0559)	0.008	−0.3519	−0.1569 (0.0541)	0.005	-	-	-	-	-	-
Average leg strength/BMI	-	-	-	-	-	-	−0.4315	−4.7901 (1.3656)	0.001	−0.4230	−4.7737 (1.3000)	0.001
Age	0.3026	0.3022 (0.1285)	0.023	0.2934	0.2932 (0.1212)	0.019	0.2649	0.2647 (0.1247)	0.039	0.2683	0.2681 (0.1170)	0.026
Vitamin B12	−0.0492	−0.0019 (0.0051)	ns	-	-	-	−0.0810	−0.0030 (0.0049)	ns	-	-	-
Vitamin D	−0.1251	−0.0298 (0.0301)	ns	-	-	-	−0.0901	−0.0214 (0.0292)	ns	-	-	-
Homocysteine	−0.0736	−0.0416 (0.0730)	ns	-	-	-	−0.0850	−0.0481(0.0701)	ns	-	-	-

The average leg strength (corrected for BMI) and the average corrected grip strength were individually analysed in two linear regression models.

- Model 1: Regression on gender, age, vitamin B12, folate, vitamin D and homocysteine.
- Model 2: Regression on gender and folate.

Fast-gait speed and TUG test results were analysed in two further linear regression models.

- Model 1′: Regression on age, vitamin B12, vitamin D, homocysteine, leg strength (corrected and uncorrected for BMI).
- Model 2′: Regression on age and leg strength (corrected and uncorrected for BMI).

In Models 1′ and 2′, gender and folate were not included as predictive variables, to avoid collinearity with the leg-strength measures.

The data were analysed using STATA 13.0 statistical software with two-sided tests, with *P* < 0.05 taken as indicating significance.

## Results

### Baseline characteristics

The baseline characteristics of men and women in the study are shown in Table 1. Men had significantly higher baseline physical and anthropometric measurements than women (*P* < 0.001). Twenty-nine per cent of men consumed at least one unit of alcohol per day whereas, as expected, none of the women consumed any (*P* < 0.01). Women were more likely to consume calcium and vitamin D supplements than men (*P* < 0.05). However, women’s baseline vitamin D levels (63.6 ± 17.7 nmol/l) were similar to men’s (63.1 ± 20.5 nmol/l), and women had a (non-significantly) greater prevalence of vitamin D deficiency (32 %) than men (18 %).

Table 1 also shows the baseline characteristics of patients with and without folate deficiency. 36 % of patients with folate deficiency had been taking supplements containing vitamin B12 compared to just 7 % of patients with normal folate (*P* < 0.05).

**Fig. 1:**
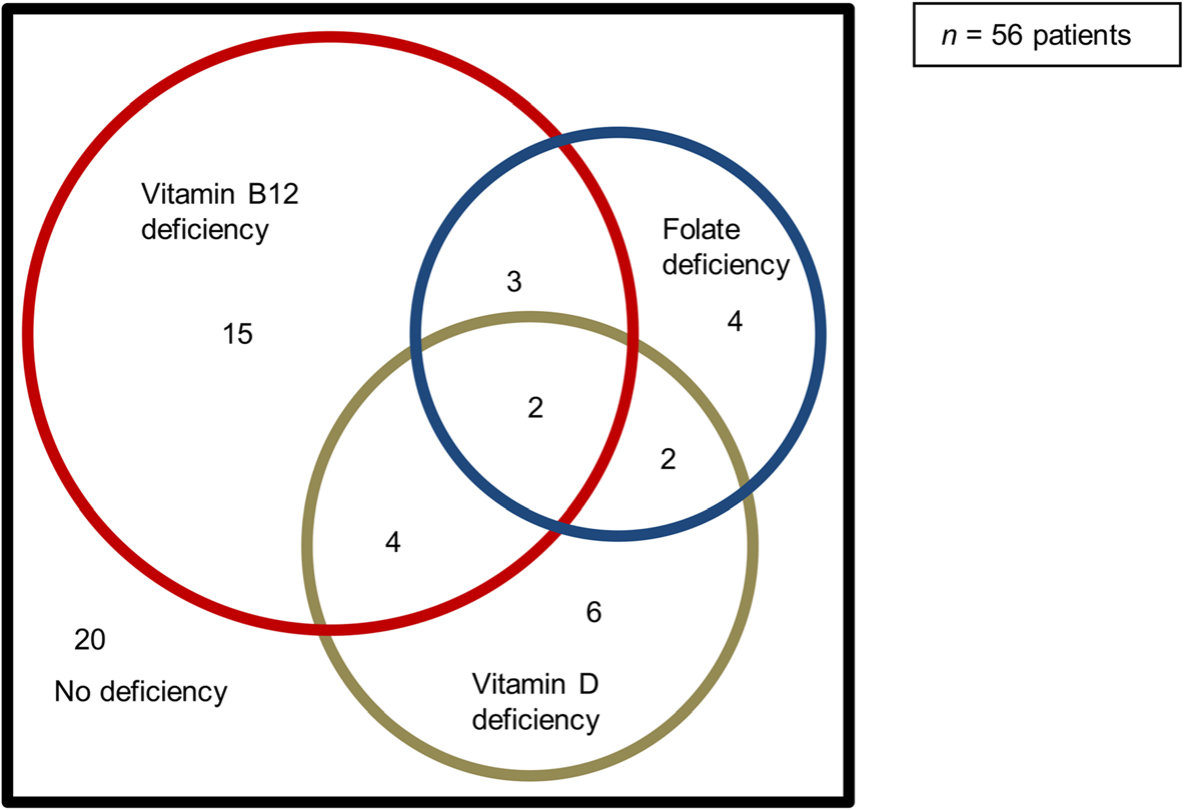
Venn diagram of the deficiencies in vitamin B12, folate, and vitamin D (*n* = 56 patients)

Vitamin B12 deficiency was present in 43 % of patients, folate deficiency in 20 %, and both deficiencies in 9 %. Hyperhomocysteinaemia was present in 52 % of patients, vitamin D deficiency in 25 % and vitamin D insufficiency in 48 % (Fig. 1). No significant differences were seen between the genders for these measures.

### Bivariate comparisons

Women with folate deficiency had significantly lower (*P* < 0.05) average leg strength (with and without correction for BMI) and average grip strength (*P* < 0.01) than women with normal folate (Table 3). Folate status did not affect male strength, but men with hyperhomocysteinaemia had significantly lower average leg strength (*P* < 0.05) and average grip strength (*P* < 0.01), with and without BMI correction.

**Table 3:** Associations between levels of vitamin B12, folate, vitamin D and homocysteine and measures of muscle strength, gait, balance and neuropathy

	Vitamin B12 deficiency (<150 pmol/l) (*n* = 24; men = 11; women = 13)	Vitamin B12 normal (≥150 pmol/l) (*n* = 32; men = 17; women = 15)	*P* value	Folate deficiency (<13.5 nmol/l) (*n* = 11; men = 4; women = 7)	Folate normal (≥13.5 nmol/l) (*n* = 45; men = 24; women = 21	*P* value	25-OH-vitamin D deficiency (<49.9 nmol/l) (*n* = 14; men = 5; women = 9)	25-OH-vitamin D normal (≥49.9 nmol/l) (*n* = 42; men = 23; women = 19)	*P* value	Hyperhomo-cysteinaemia (≥15.0 μmol/l) (n = 29; men = 15; women = 14)	Homocysteine normal (<15.0 μmol/l) (*n* = 27; men = 13; women = 14)	*P* value
Average dominant-leg strength (kgf) (both genders)	21.50 ± 8.40	22.70 ± 11.02	ns	17.22 ± 11.6	23.4 ± 9.2	ns, (0.06)	20.36 ± 7.05	22.78 ± 10.7	ns	20.22 ± 6.21	24.29 ± 12.55	ns
Average non-dominant-leg strength (kgf) (both genders)	21.64 ± 12.0	21.84 ± 10.60	ns	19.39 ± 14.58	22.33 ± 10.19	ns	18.93 ± 7.66	22.70 ± 11.95	ns	19.92 ± 6.51	23.72 ± 14.39	ns
Average leg strength (kgf) (men)	26.21 ± 2.78	27.16 ± 11.48	ns	25.76 ± 19.20	26.96 ± 10.7	ns	21.7 ± 10.5	27.90 ± 11.96	ns	22.31 ± 6.43	31.96 ± 14.4	<0.05
Average leg strength (kgf) (women)	17.63 ± 4.36	16.72 ± 5.31	ns	14.04 ± 5.14	18.18 ± 4.36	<0.05	18.49 ± 4.79	16.50 ± 4.83	ns	17.67 ± 4.19	16.62 ± 5.49	ns
Average leg strength/BMI (m^2^) (men)	1.09 ± 0.44	1.09 ± 0.49	ns	0.95 ± 0.61	1.11 ± 0.45	ns	0.92 ± 0.42	1.13 ± 0.47	ns	0.91 ± 0.24	1.30 ± 0.57	<0.05
Average leg strength/BMI (m^2^) (women)	0.74 ± 0.24	0.72 ± 0.25	ns	0.54 ± 0.22	0.79 ± 0.22	<0.05	0.73 ± 0.21	0.73 ± 0.26	ns	0.74 ± 0.23	0.72 ± 0.27	ns
Average dominant hand-grip strength (kgf) (both genders)	20.38 ± 8.45	23.23 ± 7.70	ns	19.99 ± 10.84	22.50 ± 7.32	ns	21.02 ± 8.69	22.34 ± 7.94	ns	20.05 ± 5.26	24.11 ± 9.97	ns, (0.06)
Average non-dominant hand-grip strength (kgf) (both genders)	19.32 ± 7.64	20.10 ± 7.54	ns	18.76 ± 10.60	20.01 ± 6.70	ns	20.26 ± 7.26	19.60 ± 7.68	ns	18.37 ± 5.04	21.27 ± 9.37	ns
Average grip strength (kgf) (men)	23.52 ± 9.67	26.86 ± 6.24	ns	30.39 ± 10.38	24.74 ± 7.23	ns	28.43 ± 7.06	24.92 ± 7.93	ns	21.34 ± 4.79	30.40 ± 7.85	<0.001
Average grip strength (kgf) (women)	16.75 ± 3.91	15.78 ± 2.69	ns	13.07 ± 2.56	17.28 ± 2.82	<0.01	16.31 ± 3.62	16.19 ± 3.22	ns	16.93 ± 3.62	15.53 ± 2.87	ns
Average grip strength/BMI (m^2^) (men)	0.97 ± 0.33	1.08 ± 0.34	ns	1.12 ± 0.29	1.03 ± 0.34	ns	1.18 ± 0.30	1.00 ± 0.34	ns	0.87 ± 0.22	1.24 ± 0.33	<0.01
Average grip strength/BMI (m^2^) (women)	0.71 ± 0.24	0.67 ± 0.14	ns	0.51 ± 0.14	0.75 ± 0.17	<0.01	0.64 ± 0.16	0.71 ± 0.21	ns	0.67 ± 0.15	0.72 ± 0.23	ns
Michigan Neuropathy Screening Instrument (history: maximum severity of 13 points)	2.04 ± 1.71)	1.72 ± 1.35	ns	2.18 ± 1.66	1.78 ± 1.48	ns	1.79 ± 1.12	1.88 ± 1.62	ns	2.00 ± 1.65	1.70 ± 1.35	ns
Michigan Neuropathy Screening Instrument (physical assessment: maximum severity of 10 points)	2.10 ± 1.62)	2.73 ± 1.56	ns	3.27 ± 1.49	2.27 ± 1.58	ns, (0.06)	2.57 ± 1.33	2.43 ± 1.90	ns	2.19 ± 1.52	2.76 ± 1.66	ns
Neurothesiometer VPT score right foot (V)	13.73 ± 6.01	11.8 ± 4.39	ns	15.11 ± 5.90	12.02 ± 4.88	ns	12.86 ± 4.87	12.55 ± 5.34	ns	14.18 ± 6.21	10.96 ± 3.13	<0.05
Neurothesiometer VPT score left foot (V)	15.50 ± 8.44	13.09 ± 6.42	ns	17.30 ± 8.17	13.34 ± 7.05	ns	15.32 ± 7.25	13.72 ± 7.46	ns	14.93 ± 8.08)	13.26 ± 6.59)	ns
Neurothesiometer VPT score average (V)	14.61 ± 6.95	12.44 ± 4.84	ns	16.21 ± 6.34	12.68 ± 5.61	ns	14.09 ± 5.77	13.14 ± 5.97	ns	14.55 ± 6.71)	12.11 ± 4.63)	ns
History of having fallen at least once in the preceding year	7 (Yes)	7 (Yes)	ns	5 (Yes)	9 (Yes)	ns	4 (Yes)	10 (Yes)	ns	9 (Yes)	5 (Yes)	ns
	17 (No)	25 (No)		6 (No)	36 (No)		10 (No)	32 (No)		20 (No)	22 (No)	
	29 % (Yes)	22 % (Yes)		45 % (Yes)	20 % (Yes)		29 % (Yes)	24 % (Yes)		31 % (Yes)	19 % (Yes)	
Barthel Index of Activity of Daily Living (maximum score of 20)	19.62 ± 1.05	19.78 ± 0.49	ns	19.09 ± 1.37	19.87 ± 0.46	<0.01	19.64 ± 0.84	19.74 ± 0.77	ns	19.82 ± 0.48)	19.62 ± 0.98	ns
	3 (<20)	6 (<20)		5 (<20)	4 (<20)	<0.05 (Fish)	3 (<20)	6 (<20)		5 (<20)	4 (<20)	
	21 (=20)	26 (=20)		6 (=20)	41 (=20)		11 (=20)	36 (=20)		24 (=20)	23 (=20)	
	13 % (<20)	19 % (<20)		45 % (<20)	9 % (<20)		21 % (<20)	14 % (<20)		17 % (<20)	15 % (<20)	
Lawton Instrumental Activities of Daily Living (maximum score of 8)	7 (<8)	4 (<8)	ns	5 (<8)	6 (<8)	<0.05 (Chi)	4 (<8)	7 (<8)	ns	9 (<8)	2 (<8)	<0.05 (Fish)
	17 (=8)	28 (=8)		6 (=8)	39 (=8)	<0.05 (Fish)	10 (=8)	35 (=8)		20 (=8)	25 (=8)	
	29 % (<8)	14 % (<8)		45 % (<8)	13 % (<8)		29 % (<8)	17 % (<8)		31 % (<8)	7 % (<8)	
Performance-Oriented Mobility Assessment–Balance (POMA B) (maximum score of 16)	6 (<16)	10 (<16)	ns	6 (<16)	10 (<16)	<0.05 (Chi)	6 (<16)	10 (<16)	ns	9 (<16)	7 (<16)	ns
	18 (=16)	22 (=16)		5 (=16)	35 (=16)	ns, (Fish)	8 (=16)	32 (=16)		20 (=16)	20 (=16)	
	25 % (<16)	31 % (<16)		55 % (<16)	22 % (<16)		43 % (<16)	24 % (<16)		31 % (<16)	26 % (<16)	
Performance-Oriented Mobility Assessment–Gait (POMA G) (maximum score of 12)	8 (<12)	9 (<12)	ns	6 (<12)	11 (<12)	ns, (0.05),(Chi)	5 (<12)	12 (<12)	ns	8 (<12)	9 (<12)	ns
	16 (=12)	23 (=12)		5 (=12)	34 (=12)	ns, (Fish)	9 (=12)	30 (=12)		19 (=12)	20 (=12)	
	33 % (<12)	28 % (<12)		55 % (<12)	24 % (<12)		36 % (<12)	29 % (<12)		30 % (<12)	31 % (<12)	
Timed up-and-go test (s) (average of two trials) (both genders)	11.02 ± 5.02	10.01 ± 4.16	ns	14.17 ± 6.29	9.53 ± 3.52	<0.01	11.63 ± 6.37	10.05 ± 3.74	ns	10.70 ± 4.66	10.17 ± 4.45	ns
Timed up-and-go test (s) (average of two trials) (men)	9.46 ± 2.67	9.56 ± 2.84	ns	11.59 ± 3.86	9.18 ± 2.43	ns	9.91 ± 3.98	9.44 ± 2.48	ns	9.52 ± 2.40	9.54 ± 3.16	ns
Timed up-and-go test (s) (average of two trials) (women)	12.33 ± 6.19	10.52 ± 5.34	ns	15.65 ± 7.17	9.93 ± 4.48	<0.05	12.58 ± 7.43	10.78 ± 4.83	ns	11.97 ± 6.11	10.75 ± 5.45	ns
6-m fast-gait speed (m/s) (average of two trials) (both genders)	1.07 ± 0.31	1.12 ± 0.26	ns	0.88 ± 0.30	1.15 ± 0.25	<0.01	1.03 ± 0.32	1.12 ± 0.27	ns	1.07 ± 0.28	1.12 ± 0.28	ns
6-m fast-gait speed (m/s) (average of two trials) (men)	1.19 ± 0.29	1.16 ± 0.27	ns	1.00 ± 0.28	1.20 ± 0.27	ns	1.10 ± 0.28	1.19 ± 0.28	ns	1.17 ± 0.25	1.17 ± 0.31	ns
6-m fast-gait speed (m/s) (average of two trials) (women)	0.97 ± 0.29	1.06 ± 0.26	ns	0.81 ± 0.31	1.09 ± 0.23	<0.05	1.00 ± 0.34	1.03 ± 0.24	ns	0.97 ± 0.29	1.07 ± 0.26	ns

Values are the mean ± SD or number (%) unless otherwise indicated

*VPT* vibration perception threshold

*Chi* Chi-square test

*Fish* Fisher’s exact test

*ns* Not statistically significant

On the history component of the MNSI, 44 patients (79 %) reported having muscle cramps in the legs and/or feet, and 16 patients (29 %) reported feeling weak all over most of the time. No significant association was found between these statements and levels of vitamins and homocysteine. Folate deficiency was associated with a consistent, but nonsignificant, trend towards poorer scores in both the history and physical assessment components of the MNSI.

Neurothesiometer readings were consistently (but non-significantly) lower in patients with vitamin deficiencies or hyperhomocysteinaemia than in individuals with normal levels. Similarly, the proportion of individuals who reported having fallen at least once in the preceding year was consistently (but non-significantly) higher in the presence of vitamin deficiencies or hyperhomocysteinaemia than with normal levels. Logistic regression of a positive fall history in the preceding year with levels of vitamin D, vitamin B12, folate and homocysteine did not reveal any significant associations. However, logistic regression demonstrated that average leg strength had a significant association with the risk of a positive fall history (odds ratio (OR) = 0.89 per 1 kgf increase in uncorrected leg strength, 95 % CI 0.80–0.98, *P* < 0.05; OR = 0.12 per 1 m^2^ increase in leg strength corrected for BMI, 95 % CI 0.02–0.92, *P* < 0.05). Thus the greater the leg strength, the lower the odds of having a positive fall history. The OR and statistical significance were minimally affected when controlling for age and gender (data not shown).

Patients with folate deficiency had a significantly lower mean BADL score than patients with normal folate levels (19.09 ± 1.37 versus 19.87 ± 0.46, *P* < 0.01). Similarly, 45 % of folate-deficient patients had a less-than-perfect BADL score, compared with only 9 % of folate-sufficient patients (*P* = 0.01 by Fisher’s exact test). Similar negative effects of folate deficiency on the proportion of patients with perfect scores were seen in POMA B (*P* < 0.05) and POMA G (*P* = 0.05), with chi-square tests. Patients with folate deficiency and those with hyperhomocysteinaemia were also less likely than those with normal levels to have a perfect score in the IADL (*P* < 0.05 for both chi-square and Fisher’s exact tests) (Table 3).

When patients were analysed according to gender, folate deficiency had a significant (*P* < 0.05) negative effect on 6-m fast-gait speeds and TUG times in women, but not in men. Combining men and women in the analysis increased the level of significance for this effect (*P* < 0.01 for both measures by *t*-test). No significant association between these variables was demonstrated by regression analysis (data not shown). No significant effect or trend was observed between vitamin and homocysteine status and the results of sway-area analyses (data not shown).

### Regression analyses

Regression analyses were performed to identify variables associated with leg strength and grip strength. The values of leg strength and grip strength corrected for BMI had a kurtosis of 3.17 and 2.72, respectively, and a positive skew of 0.69 and 0.78, respectively. Regression analyses were performed with these values both untransformed and natural-log-transformed. Value transformation did not affect significance, and untransformed values were used in the statistical analyses.

**Fig. 2:**
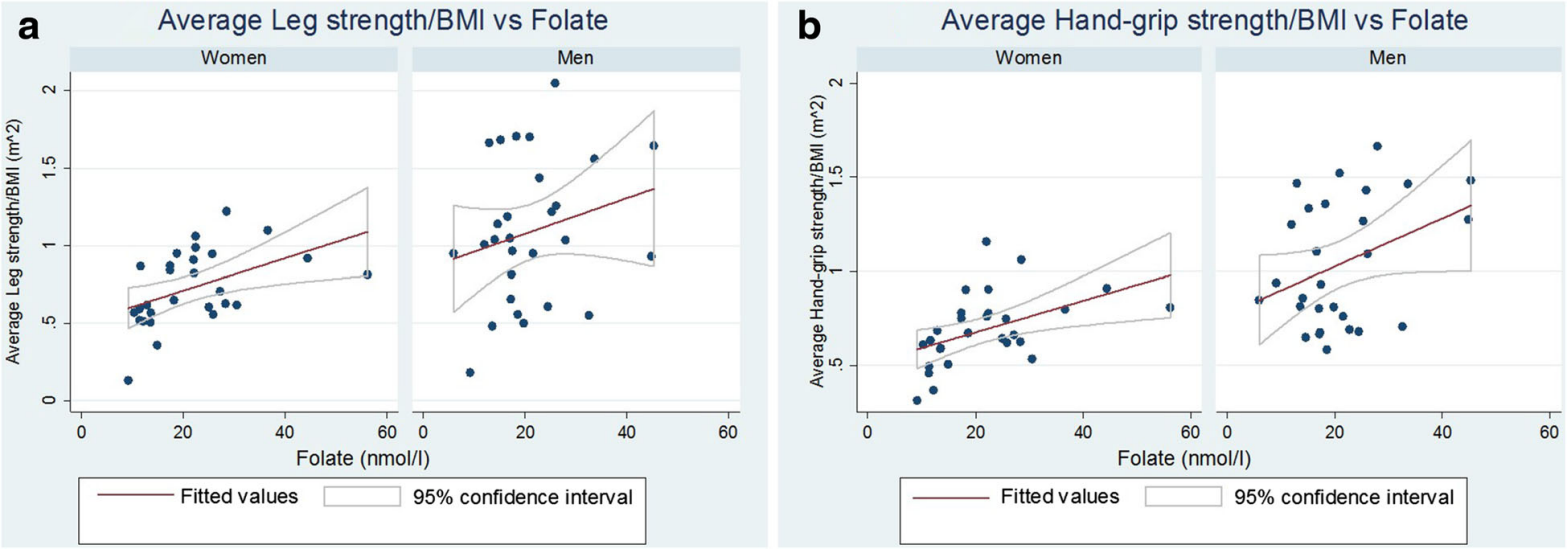
Serum levels of folate and measures of strength in individual patients. For each of the 28 female and 28 male participants, serum folate was plotted against **a** the average leg strength (corrected for BMI) and **b** the average hand-grip strength (corrected for BMI)

Two regression models were tested for the association of leg strength and grip strength (both corrected for BMI) with patient characteristics. Model 1 included multiple variables, of which gender predicted leg strength (*P* = 0.001) and grip strength (*P* < 0.001), and folate predicted grip strength (*P* = 0.017). Model 2 only included gender and folate levels, both of which predicted average leg strength (F(2,53) = 9.79, *P* < 0.001, *R* ^2^ = 0.27) and grip strength (F(2,53) = 17.74, *P* < 0.001, *R* ^2^ = 0.40) (Table 2, Fig. 2).

Two further regression models were tested for the association of variables with 6-m fast-gait speed and TUG results. Model 1′ included multiple variables, of which leg strength (both uncorrected and corrected for BMI) and age predicted both fast-gait speed and TUG time. Model 2′ only included leg strength (corrected and uncorrected values were analysed separately) and age, both of which predicted fast-gait speed and TUG time (Table 2).

## Discussion

**Fig. 3:**
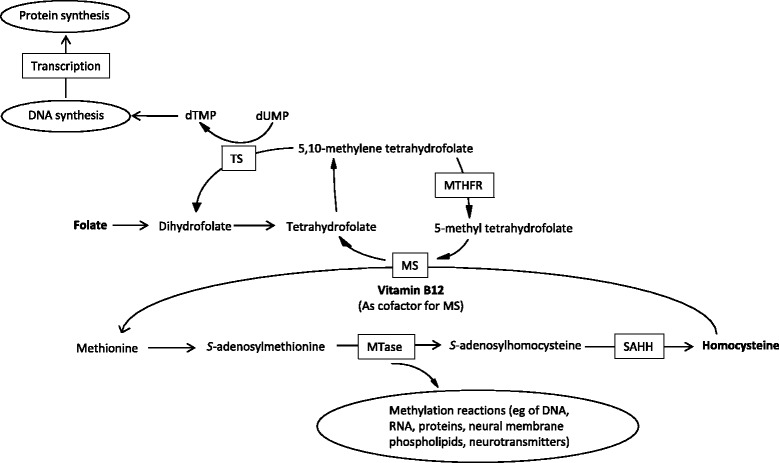
Functional relationships between vitamin B12, folate and homocysteine. dUMP, deoxyuridine monophosphate; dTMP, deoxythymidine monophosphate; MS, methionine synthase; MTase, methyltransferase; MTHFR, methylenetetrahydrofolate reductase; SAHH, *S*-adenosylhomocysteine hydrolase; TS, thymidylate synthase

Vitamin B12 and folate are involved in the conversion of homocysteine to methionine (Fig. 3), and hyperhomocysteinaemia is a marker for vitamin B12 deficiency. Many factors that predispose an individual to vitamin B12 deficiency also predispose to vitamin D deficiency. These factors include meat-deficient diets, malabsorption and old age. Among 56 patients >65 years old with diabetes mellitus at a polyclinic in Singapore, we found a high prevalence of deficiencies of vitamin B12, folate and vitamin D, as well as hyperhomocysteinaemia.

In our cohort, more women than men took calcium and vitamin D supplements (Table 1), presumably because of greater awareness of the need for the prevention of osteoporosis in women [54, 55]. These supplements typically supply 200–400 IU of vitamin D3 per day in combination with calcium carbonate. Despite this level of supplementation, mean levels of vitamin D were very similar in men and women. However, almost twice as many women as men were vitamin D deficient, although this difference was not statistically significant. In a study of 134 inpatients ≥65 years old in a rehabilitation centre in Singapore, although 26.9 % reported taking calcium and/or vitamin D supplementation, 44 % were vitamin D deficient, and a further 41.8 % were vitamin D insufficient, with no significant difference in vitamin D levels between those who were taking vitamin D supplementation and those who were not [6]. These results demonstrate the need for future studies of the effects of age and diabetes mellitus on the bioavailability of vitamin D supplements.

Folate deficiency was significantly associated with vitamin B12 supplementation (*P* < 0.05). It is possible that supplementation with relatively high doses of vitamin B12, a common clinical practice, could drive up folate metabolism and hence lead to folate deficiency (Fig. 3). Indeed, five out of the seven patients in our cohort who had vitamin B12 supplementation had been prescribed high doses of vitamin B12 in the form of either regular intramuscular cyanocobalamin, oral methylcobalamin or oral vitamin B12 in combination with vitamins B1 and B6 (eg Neurobion®) (data not shown). Future studies of the effect of vitamin B12 supplementation on folate deficiency are needed.

To determine muscle strength, the convention is to use the maximum dominant-limb measure from three attempts. However, in our cohort, the average grip strength and the average leg strength were measured instead, as we believed, on a theoretical basis, that it would provide a better assessment of overall strength by minimizing the effect of unilateral limb pathology (such as a pre-existing limb injury, severe osteoarthritis in the dominant limb or occupational-use preference leading to an unusually strong limb) that could skew the results. Moreover, the maximal dominant-leg strength and maximal dominant-grip strength were separately analysed in the sensitivity analyses, and the results were similar to those obtained with average-strength measurements (data not shown).

Folate levels were not significantly correlated with unadjusted leg-strength or grip-strength measures, and the correlation was only evident when strength was corrected for BMI (i.e. with BMI as the denominator) and when men and women were analysed separately. This correction is justified because, with all else being equal (including height), a person with a greater body mass and habitus is typically stronger than one with a lower body mass [43, 56]. The analysis was, therefore, designed to determine associations with strength, rather than habitus. Many studies of strength measures do not make this adjustment, with the possible result that significant associations could have been missed.

The coefficients for the regression of folate on corrected leg strength and on corrected grip strength were both 0.01 (Table 2), so that a difference of 10 nmol/l in folate measurements would be associated with a variation in leg strength or grip strength of BMI × 0.01 × 10. In two women with BMI of 24 kg/m^2^, each 10 nmol/l difference in folate would represent 2.4 kgf difference in strength. This linear association was valid for the full range of folate measurements, which extended from 6.0 to ≥56.2 nmol/l (56.2 nmol/l was the upper limit of the folate assay), with an overall mean of 21.5 ± 10.04 nmol/l. The correlation of folate with strength had a greater variance in men than in women (Fig. 2), possibly reflecting sexual dimorphism or differences in occupation and recreational activities, and their accompanying physical requirements. Although no significant association was found between folate and strength in men in our pilot study, this relationship should be studied further with a larger sample size.

Corrected or uncorrected leg strength was, in turn, negatively associated with the probability of having fallen in the preceding year. With an OR of 0.89 for uncorrected leg strength, a difference of 1 kgf would be associated with an 11 % difference in the probability of having fallen (and 2.4 kgf, as discussed in the preceding paragraph, with 1–0.89^2.4^ = 24 % probability difference).

Vitamin D and vitamin B12 deficiencies and hyperhomocysteinaemia, as presented in the Background section, have biologically plausible mechanisms that could account for an observed reduction of muscle strength. In our cohort, trends toward poorer performance in strength measurements, gait measurements, neurothesiometer scores and history of falls were seen in patients with deficiencies in vitamin B12, vitamin D and folate and with hyperhomocysteinaemia (Table 3). Whilst only folate deficiency and hyperhomocysteinaemia yielded significantly poorer scores in any of their respective measurements, the effects of folate deficiency and hyperhomocysteinaemia seem to be mediated through different pathways. For instance, hyperhomocysteinaemia affected strength in men, whereas folate deficiency had a greater effect on women than on men. We suggest that the effect of folate deficiency on strength and gait measurements involves biological mechanisms beyond the deleterious properties of homocysteine per se. These mechanisms could include folate-specific activities [57], such as neurotransmitter synthesis, myelination, synthesis of DNA and protein, DNA methylation and epigenetic regulation (Fig. 3).

Our findings concur with those of a study of 796 respondents in the Singapore Longitudinal Ageing Study, which showed that low folate and high homocysteine have independent associations with physical and functional decline as measured by POMA and IADL scores [26]. In a clinical trial [58] involving 92 patients in an Australian residential home, 49 received multivitamins for 6 months, leading to increased levels of folate and correspondingly decreased TUG times (*r* = −0.32, *P* = 0.043), compared with untreated individuals. However, in a clinical trial in the Netherlands [59] of individuals ≥65 years old with hyperhomocysteinaemia, supplementation with folate and vitamin B12 for two years did not reduce the decline in hand-grip strength or prevent falls, relative to placebo. The characteristics of this study population differed considerably from those of our cohort. The mean height of the individuals in the intervention group, who were predominantly European, was 169.4 ± 9.4 cm, and the weight was 77.9 ± 13.3 kg, with a baseline hand-grip strength of 30.8 ± 10.6 kgf. They were only mildly hyperhomocysteinaemic (median homocysteine 14.5 μmol/l), with few vitamin B12-deficient or folate-deficient individuals. BMI was not used as a denominator for muscle-strength measures. Moreover, a clinically beneficial effect of homocysteine reduction through folate supplementation was seen on gait and on physical-performance measures in a subgroup of individuals >80 years old [59].

The baseline values for muscle/grip strength can vary between different ethnic/geographical groups. In our study cohort, the mean grip strengths were 25.54 ± 7.78 kgf in men (median 24 kgf) and 16.22 ± 3.29 kgf in women (median 16 kgf). These values are comparable to the means of 24.7 kgf for men and 16.5 kgf for women reported in a Taiwanese population aged 70–74 years old [60], and are within the thresholds of <26 kgf for men and <18 kgf for women for initiation of sarcopenia assessment in Asian populations [61]. In European populations, the cut-offs for muscle strength are much higher than in Asian populations. In a Finnish study, the hand-grip-strength cut-points to identify people ≥55 years old who were at risk of mobility limitation were 37 kgf in men (without considering BMI) and 21 kgf in women [43]. In the Italian InCHIANTI study, cut-offs for hand-grip strength of 30 kgf for men and 20 kgf for women were identified [62]. Further studies are needed to enable us to understand the role of folate deficiency in muscle weakness in Asian populations, especially where mandatory fortification of food with folate does not occur [63].

Limitations of this pilot study included the very small sample size, which carries a risk for type II errors, and the cross-sectional nature of the study, such that causality cannot be determined from the identified associations. The measurements of vitamin B12 and folate in this study might not perform as well as indicators such as red-blood-cell folate, methylmalonic acid [64] and methylenetetrahydrofolate reductase polymorphisms [65], which were not investigated. Addition of the Lord swaymeter, serum protein and albumin tests for the second phase of the study resulted in the absence of results for the 11 patients who were included in the first phase, which reduced the sample size for these assessments. Similarly, the use of different instruments to measure leg strength in the first phase and the second phase could have introduced a systematic error in leg-strength measurements between the patients in the two phases, with the potential to introduce a type I error and affect the internal validity. Indeed, the average leg strength (corrected for BMI) for the first 11 patients was 0.699 ± 0.118 m^2^, and for the subsequent 45 patients was 0.961 ± 0.060 m^2^ (*P* = 0.06). The difference most likely reflects an earlier selection bias toward recruitment of weaker patients. As only one patient could be recruited at a time and often more than one patient were simultaneously being referred for recruitment, a preference for the recruitment of the weaker-looking patient likely occurred due to the physical strain of using the hand-held dynamometer in the first phase of the study. Moreover, we did not perform a formal statistical comparison between the two dynamometers. However, some evidence suggests that the leg strength measurements were valid. The instruments were both calibrated to measure force in kgf, and the examiner was able to fully resist the knee extension of all the 11 patients in the first phase. Studies have generally shown acceptable correlation of hand-held dynamometry with standard isokinetic dynamometry [66], especially for weaker, older people [42]. The average grip strength (corrected for BMI) was measured using the same dynamometer for patients in both phases, and folate predicted corrected grip strength with a similar coefficient of regression as that with corrected leg strength. Finally, sensitivity testing using bivariate analyses for each of the phases of the study by gender showed that folate had a consistently positive but nonsignificant association with the corrected average leg strength, and that significance was attained only after combining the data from the two phases.

Affective and cognitive function were not tested formally in this study, even though these functions might be confounding variables in falls. We presumed that the recruitment process itself, involving motivation, cooperation, understanding and informed consent, would create a selection bias in favour of individuals who were not depressed or demented. However, it is possible that some of the patients included in our study did have unidentified affective or cognitive disorders.

## Conclusions

In a cohort of 56 patients >65 years old with diabetes mellitus, serum levels of folate strongly and significantly predicted muscle strength (corrected for BMI), especially in women.

The effect of folate on strength was possibly mediated less through homocysteine, and more through direct, folate-specific mechanisms (such as myelination, neurotransmitter formation, synthesis of DNA and protein and epigenetic methylation of DNA).

A clinical trial is warranted to investigate the role of folate in the aetiology of sarcopenia and falls, especially where folate deficiencies are prevalent and food fortification is not mandatory. This pilot study provides measures of the prevalence and effect size of folate deficiency, to facilitate the design of such a trial.

## Abbreviations

25-OH-vitamin D: 25-Hydroxyvitamin D
ALT: Alanine aminotransferase
ANOVA: Analysis of variance
BADL: Barthel Index of Activities of Daily Living
BMI: Body mass index
CI: Confidence interval
CIRB: Centralised institutional review board
DNA: Deoxyribonucleic acid
dTMP: Deoxythymidine monophosphate
dUMP: Deoxyuridine monophosphate
FP: Family physician
HDL: High-density lipoprotein
IADL: Instrumental Activities of Daily Living
LDL: Low-density lipoprotein
MCI: Master of Clinical Investigation, National University of Singapore
MNSI: Michigan Neuropathy Screening Instrument
MS: Methionine synthase
MTase: Methyltransferase
MTHFR: Methylenetetrahydrofolate reductase
NRF: National Research Foundation
ns: Not significant
OR: Odds ratio
POMA B: POMA-balance
POMA G: POMA-gait
POMA: Performance-Oriented Mobility Assessment
SAHH: *S*-adenosylhomocysteine hydrolase
SD: Standard deviation
SE: Standard error
SingHealth: Singapore Health Services
STATA: Data analysis and statistical software
TS: Thymidylate synthase
TUG: Timed up-and-go test
UK: United Kingdom
US: United States of America
VPT: Vibration perception threshold
